# Environmental stress and flowering time: The photoperiodic connection

**DOI:** 10.4161/psb.29036

**Published:** 2014-04-30

**Authors:** Matteo Riboni, Alice Robustelli Test, Massimo Galbiati, Chiara Tonelli, Lucio Conti

**Affiliations:** 1 Department of Biosciences; Università degli Studi di Milano; Milan, Italy; 2 Fondazione Filarete; Milan, Italy

**Keywords:** plant stress response, Florigen, drought escape, plant adaptive development, photoperiodic flowering

## Abstract

Plants maximize their chances to survive adversities by reprogramming their development according to environmental conditions. Adaptive variations in the timing to flowering reflect the need for plants to set seeds under the most favorable conditions. A complex network of genetic pathways allows plants to detect and integrate external (e.g., photoperiod and temperature) and/or internal (e.g., age) information to initiate the floral transition. Furthermore different types of environmental stresses play an important role in the floral transition. The emerging picture is that stress conditions often affect flowering through modulation of the photoperiodic pathway. In this review we will discuss different modes of cross talk between stress signaling and photoperiodic flowering, highlighting the central role of the florigen genes in this process.

## 

### Photoperiodic-Dependent Activation of Flowering

After the floral transition the shoot apical meristem (SAM) changes its identity switching from vegetative to reproductive. In annual *Arabidopsis* ecotypes, the transition to flowering is strongly promoted by variations in day length (photoperiod). The photoperiodic pathway promotes flowering when *Arabidopsis* plants are exposed to long days (LDs) conditions (typical of spring and summer). Photoperiodic flowering is the result of complex interactions between the circadian clock (an endogenous timekeeping mechanism) and external cues, which ultimately results in the activation of a set of floral genes.^1^ Central to photoperiod-dependent flowering is the pattern of accumulation of the flowering protein CONSTANS (CO).^2^^-^^4^ *CO* expression is regulated transcriptionally by the circadian clock through the GIGANTEA (GI)-FLAVIN-BINDING, KELCH REPEAT, F-BOX (FKF1) complex.^5^^,^^6^ LDs also promote the stabilization of CO protein at the end of a LD via activation of the photoreceptors PHYTOCROME A, CRYPTOCHROME 1 and 2 (CRY1 and 2).^3^ CO protein promotes the transcriptional activation of the florigen genes *FLOWERING LOCUS T* (*FT*) and *TWIN SISTER OF FT* (*TSF*) in the phloem companion cells.^7^^-^^10^ FT and FT-likes proteins encode small proteins with similarity to the Raf Kinase Inhibitor Proteins (RKIP). They usually act as systemic signals, since these proteins are able to move between cells.^11^ FT protein moves from the leaves to the SAM where it interacts with the SAM-specific bZIP transcription factors FLOWERING LOCUS D (FD) and FD PARALOG (FDP) to initiate the floral transition.^12^^-^^16^ Here, the FT/FD heterodimer activates several MADS box-type transcription factors, namely *SUPPRESSOR OF OVEREXPRESSION OF CONSTANS 1* (*SOC1*), *APETALA1*, and *FRUITFUL*, responsible for triggering the floral transition.^17^^,^^18^

Florigen gene expression has been demonstrated to play a pivotal role in photoperiodic flowering in different plants including *Arabidopsis*, a facultative LD plant and Rice (*Oryza sativa*), a facultative short day (SD) plant.^19^ However, florigen expression is not always dependent upon photoperiod variations as in the case of the day neutral plant Tomato (*Solanum lycopersicum*).^20^ This implies that florigen upregulation can also occur in response to internal or external stimuli other than variations in day length. The data reviewed here reinforces the idea that the photoperiodic pathway and the florigen genes are central nodes of a wider network receiving a multitude of external inputs. Furthermore, mechanisms that couple photoperiodic flowering with stress acclimation are emerging.

### Stress-Dependent Activation of *FT* Expression

LDs promote flowering via activation of the florigen genes in *Arabidopsis*. However, it is now apparent that the *FT* promoter conveys several environmental information, in some cases independent of day length. Many plant species are induced to flower following drought stress which results in a drought escape response - DE -.^21^^-^^27^ The onset of DE maximizes the chances to set seeds, thus “escaping” from a potentially lethal drought condition.^28^ We have recently shown that in *Arabidopsis* DE occurs under LDs but not SDs, thus revealing a strong interdependence of certain drought responses on photoperiod. Genetic screens showed that photoperiod-stimulated GI activity is necessary and sufficient to trigger a drought dependent activation of the florigen genes *FT* and *TSF*.^29^

The phytohormone ABA plays a pivotal role in mediating several drought adaptive mechanisms although its precise role in flowering is still poorly understood.^30^ Genetic and expression data suggest a role for ABA in DE response, through the activation of the florigen genes.^29^ *aba1* mutants are impaired in ABA biosynthesis and display reduced accumulations of *FT* and *TSF* transcripts, especially under drought conditions. In addition to *FT* and *TSF* another *FT*-like genes *MOTHER OF FT AND TFL1* (*MFT*) all appear to be positively regulated by ABA.^31^^,^^32^ Taken together these data argue in favor for a positive role for endogenous ABA in flowering via potentiation of florigen-like genes in a photoperiodic manner.

Some plants use drought stress as a primary cue to flowering. Recent studies suggest that drought stress is involved in the upregulation of the florigen genes in the tropical tree *Shorea beccariana*.^33^ Moderate increases in drought index promote an increase of *SbFT* transcript accumulations early in bud development, preceding flower morphological changes. *Shorea beccariana* grows at the equator where day length and temperature are constant throughout the year. It is thus plausible that drought spells could represent a major external cue to trigger mass flowering in this species via direct activation of *FT* independent of photoperiod. Photoperiod-independent modes of activation of *FT* exist also in *Arabidopsis* where an increase in ambient temperature is reflected in augmented *FT* transcript accumulation.^34^ A key component of this mechanism is the bHLH transcription factor PHYTOCHROME INTERACTING FACTOR 4 (PIF4) directly activating *FT* expression largely independent of CO.^35^ It is intriguing to note that occurrence of drought episodes often coincides with an increase in ambient temperature, at least in temperate climates. Whether ambient temperature also plays a regulatory role in DE response is thus an interesting question.

Unlike the thermosensory pathway, the mechanism through which drought stimuli affect *FT* activation is unknown. Drought stress results in an increase in *FT* expression with no evident effect on the physiological circadian oscillation of *FT*.^29^^,^^36^ Because the pattern of *FT* transcript accumulation depends on variations in CO protein, drought might directly affect *CO* expression. FLOWERING BHLH 1 (FBH1), a *CO* positive activator, is phosphorylated in vivo following ABA signaling activation.^37^^,^^38^ Although the precise role of phosphorylation on FBH1 protein function is still unknown, this finding could support a role for ABA in *CO* transcription under drought conditions. Also, EID1-like protein 3 (EDL3), a positive regulator of ABA signaling is an activator of *CO*. *EDL3* transcript is upregulated following ABA applications.^39^ Although these findings point to a link between ABA and photoperiodic flowering via *CO* transcript accumulations we could find only minor variations in *CO* transcript in wild-type or *aba1* mutant plants subjected to drought stress (Fig. 1A).

**Figure:**
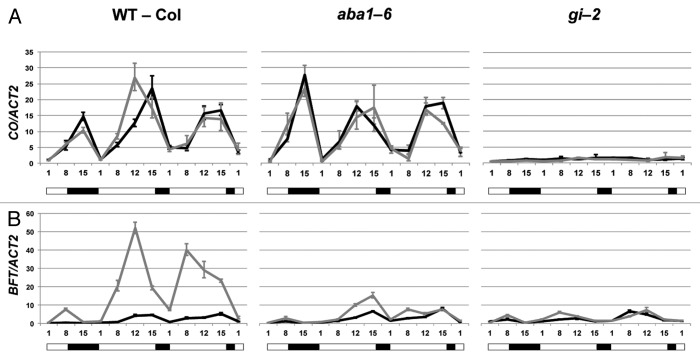
**Figure 1.** Real-time qPCR of *CO* (**A**) or *BFT* (**B**) transcripts in 3 wk-old wild-type (Col-0), *aba1-6* or *gi-2* seedlings. Plants were subjected to normal watering (black lines) or reduced watering (gray lines) regimes and harvested at the indicated time points in coincidence with the light phase (open bar) or in the dark (black bar) during a SDs to LDs shift. At each time point, values represent fold change variations of *CO* or *BFT* transcript levels relatively to Col-0 under NW. *ACT2* expression was used for normalization; error bars represent SD of 2 technical replicates. A representative experiment of 2 biological replicates is shown.

Drought (via ABA) could affect CO protein activity or stability. For example, besides the well-established role in seed germination the ABA signaling protein ABA INSENSITIVE 3 (ABI3) is involved in the control of flowering time. *abi3* mutants are early flowering under both SDs and LDs while *ABI3* overexpression results in an increased vegetative phase under LDs.^40^ ABI3 binds to the CO CCT (CO, CO-like, TOC1) domain involved in the recruitment of the CO protein to the promoter of *FT*.^41^^,^^42^ Thus, interaction with ABI3 may interfere with CO (and perhaps other CCT domain - containing proteins) binding to DNA. Intriguingly, following spray with ABA, *abi3* mutants display high levels of *TSF*, suggesting a repressive role for ABI3 on *TSF* expression.^43^ In germinating seeds, the expression of *MFT* is downregulated by ABI3.^31^ ABI3 may thus act as a negative regulator of flowering through downregulation of florigen-like genes.

Despite the GI-CO module being responsible for most of the activation of *FT*, *FT* upregulation may occur independently of either CO or GI. For example, warm temperatures results in an acceleration of flowering in the absence of GI and CO activities.^34^ In contrast, a DE response can be induced in *co* but not *gi* mutants, although it is unknown whether drought can stimulate *FT* upregulation in the absence of CO activity.^29^ Nonetheless this observation suggests that drought signals can overcome CO action to trigger flowering, provided that GI is photoperiod-stimulated. In support of the key role of GI in DE, ABA hypersensitive mutants are early flowering under LDs, but not under SDs. Thus ABA hyper-activation cannot override the requirement of photoperiod-stimulated GI in flowering.^29^ Examples of GI dependent but CO-independent mechanisms for *FT* activation have been described.^35^^,^^44^^-^^48^ However it is currently unclear how drought might affect GI-derived signals upon the *FT* promoter. Other pathways could facilitate the responsiveness of *FT* to photoperiod-stimulated GI. For example, similarly to *gi*, *cry2* mutants have a defective DE response, despite constitutively accumulating increased ABA levels compared with wild type.^29^^,^^49^ Therefore, one could speculate that also CRY2 may participate in the GI- and ABA-dependent activation of *FT*.

*Arabidopsis* has 3 florigen genes, of which 2 (*FT* and *TSF*) act redundantly to mediate photoperiodic flowering.^8^^,^^50^^,^^51^ Despite this functional redundancy, *FT* and *TSF* transcripts are found in a non-overlapping pattern of expression.^8^ Also, *TSF* expression (but not *FT*) can be activated under SDs following exogenous applications of a synthetic Cytokinin (CK).^52^ Thus, unlike ABA, CKs do not require a photoperiodic input for the activation of *TSF*. Because of this reduced dependence on photoperiod, *TSF* upregulation might also occur in the absence of CO (although still in a GI-dependent manner) under drought conditions and contribute to the DE response observed in *co* mutants. In conclusion, more work is needed to clarify the mode of *FT* and *FT-like* genes activations under drought conditions and their specific interdependence with the photoperiodic pathway machinery.

### Stress Dependent Downregulation of *FT* Expression

Not all abiotic stresses are interpreted as an escape signal. For example, cold stress delays flowering and alters the diurnal oscillation of *FT* expression even under inductive photoperiodic conditions. It has been shown that cold temperatures induce the degradation of CO protein via an ubiquitin/proteasome pathway that involves the E3 ubiquitin ligase HIGH EXPRESSION OF OSMOTICALLY RESPONSIVE GENE 1 (HOS1).^53^ Under normal growth temperature HOS1 acts as a general component of photoperiodic flowering by destabilizing CO protein in response to daylight signals.^54^ Modulation of HOS1 activity by light and cold temperature plays a crucial role in the daily pattern of CO accumulation, thus revealing yet another example of interplay between environmental cues and day length perception via florigen regulation.

A different osmotic stress, salinity, delays flowering in *Arabidopsis* by interfering with the photoperiodic pathway. Interestingly salt affects *FT* at 2 different levels, transcriptional and post-transcriptional. Salt stress promotes GI protein degradation through an unknown ubiquitin/proteasome pathway.^55^ Consequently, salt negatively regulates *CO* and *FT* transcripts accumulation. Salt stress delays flowering by activating the floral repressor *BROTHER OF FT* (*BFT*), a florigen-like protein with opposite function to *FT*.^56^ BFT competes with FT for the binding to FD, thus delaying the switch to flowering. *BFT* is strongly responsive to drought stress and ABA.^57^ We also confirmed that *BFT* can be transcriptionally activated under drought conditions in an ABA dependent manner and this regulation is dependent on GI (Fig. 1B). Thus, *BFT* expression is subject to a similar regulatory mechanism that orchestrates the activation of *FT* and *TSF* and is responsible for the DE response. However, the physiological role of *BFT* in DE is unclear since under drought conditions the positive regulation of flowering (i.e., via *FT* and *TSF*) clearly prevails over *BFT*. One could hypothesize that the balance between florigen and anti-florigen proteins is necessary to generate an optimal duration of reproductive development according to environmental stress. In this sense BFT may buffer FT action and prevent a premature interruption of inflorescence development. Deciphering the regulatory logic of the different florigen genes is thus an important goal to gain insights into the different flowering adaptations to stress as well as the mechanisms that govern crop seed yield under adverse conditions.

### Future challenges: coordination of escape and tolerance strategies

A question arise as to how plants might coordinate flowering networks with tolerance responses, which allow individual cells to survive under stress conditions. GI is emerging as a key node connecting different abiotic responses with flowering time. *gi* mutants display different phenotypes including an increased salt tolerance.^55^ GI directly binds to SALT OVERLY SENSITIVE 2 (SOS2) protein and prevents its action under normal growth condition. Salt stress triggers the degradation of GI, thus releasing SOS2 and activating a salt-stress tolerance pathway. Besides salt, GI affects several developmental transitions (e.g., seedling photomorphogenesis and flowering time) as well as different environmental responses (starch accumulation, sucrose metabolism, sensitivity to light and oxidative stress).^48^^,^^58^^-^^62^ Furthermore GI controls guard cell activity.^63^ GI could coordinate different responses through a process of sequestration and release of interacting partners.^55^ In this model GI stability plays a key role through which plants can coordinately regulate independent processes with flowering.

In conclusion, plant adaptation to stress is complex and involves different strategies. In *Arabidopsis* the escape strategy requires a positive integration between photoperiodic and drought-dependent signals. A floral delay strategy takes place upon conditions where growth restraint provides an adaptive advantage over an escape, namely on salt.^64^ In all these cases, modulation of florigen genes represents the common central thread for how differential flowering strategies are enacted.
